# Advancing Breast Cancer Care in Patients Aged 80 and Above: A Personalized and Multidisciplinary Management to Better Outcomes

**DOI:** 10.3390/jpm15030090

**Published:** 2025-02-27

**Authors:** Maria Natale, Alba Di Leone, Domenico Fusco, Cristina Accetta, Andrea Bellieni, Beatrice Carnassale, Sabatino D’Archi, Flavia De Lauretis, Enrico Di Guglielmo, Antonio Franco, Diana Giannarelli, Stefano Magno, Francesca Moschella, Alejandro Martin Sanchez, Lorenzo Scardina, Marta Silenzi, Riccardo Masetti, Gianluca Franceschini

**Affiliations:** 1 Breast Unit, Department of Women, Children and Public Health Sciences, Fondazione Policlinico Universitario Agostino Gemelli IRCCS, Università Cattolica del Sacro Cuore, Largo Agostino Gemelli 8, 00168 Rome, Italy; alba.dileone@policlinicogemelli.it (A.D.L.); cristina.accetta@policlinicogemelli.it (C.A.); beatrice.carnassale@guest.policlinicogemelli.it (B.C.); sabatino.darchi@policlinicogemelli.it (S.D.); flavia.delauretis@gmail.com (F.D.L.); enricodiguglielmo@yahoo.com (E.D.G.); antonio.franco@guest.policlinicogemelli.it (A.F.); stefano.magno@policlinicogemelli.it (S.M.); francesca.moschella@policlinicogemelli.it (F.M.); martin.sanchez@policlinicogemelli.it (A.M.S.); lorenzo.scardina@policlinicogemelli.it (L.S.); martasilenzi@tiscali.it (M.S.); riccardo.masetti@policlinicogemelli.it (R.M.); gianluca.franceschini@policlinicogemelli.it (G.F.); 2 Department of Geriatrics and Orthopaedics, Fondazione Policlinico Universitario Agostino Gemelli IRCCS, Università Cattolica del Sacro Cuore, Largo Agostino Gemelli 8, 00168 Rome, Italy; domenico.fusco@policlinicogemelli.it (D.F.);; 3 Facility of Epidemiology and Biostatistics, Fondazione Policlinico Universitario Agostino Gemelli IRCCS, Università Cattolica del Sacro Cuore, Largo Agostino Gemelli 8, 00168 Rome, Italy; diana.giannarelli@policlinicogemelli.it

**Keywords:** breast cancer treatment, elderly patients, overeighties, geriatric assessment

## Abstract

**Background:** Breast cancer in women aged 80 years and older accounts for about 12% of cases, but its management is challenging due to the population’s heterogeneity and the lack of relevant evidence-based guidelines. Treatment decisions must consider biological age, comorbidities, life expectancy, therapy-related toxicities, and tumor biology. This study evaluates the clinical outcomes of elderly breast cancer patients treated with a multidisciplinary approach, including oncologists, surgeons, and geriatric specialists. **Materials and Methods:** A retrospective analysis of breast cancer patients aged ≥80 years treated at Fondazione Policlinico Universitario Agostino Gemelli IRCCS in Rome, Italy, from January 2016 to December 2020 was conducted. The study reviewed clinicopathological data, surgery, adjuvant therapies, and clinical outcomes. Treatment decisions were guided by multidisciplinary evaluations, including onco-geriatric assessments (GA) and guided treatment decisions. Primary outcomes included overall survival (OS), disease-free survival (DFS), and cancer-specific survival (CSS). Surgical and treatment-related complications were also documented. **Results:** A total of 238 patients aged ≥80 years were included in the study. Of these, 203 (85.3%) underwent breast-conserving surgery, while 35 (14.7%) underwent mastectomy. Axillary surgery was performed in 129 (54%) cases. Regarding adjuvant treatments, 93 (39.1%) patients received radiotherapy, and 101 (42.4%) received endocrine therapy alone. Chemotherapy was administered to six high-risk patients following GA, with no reported toxicities. Over a median follow-up of 42.3 months, the study reported one local recurrence (0.5%), one regional node recurrence (0.5%), and 19 cases of distant metastases (9%). A total of 19 patients (9%) died due to breast cancer. The overall complication rate was low, with 10% experiencing wound dehiscence, hematoma, lymphedema, or similar issues. Five-year survival outcomes were OS 73.3%, DFS 66.6%, and CSS 88.5%. **Conclusions:** This study highlights that a multidisciplinary approach to breast cancer management in patients aged ≥80 years yields favorable clinical outcomes with low recurrence, metastasis, and complication rates. The personalized treatment strategies, guided by onco-geriatric assessments, balance survival benefits with quality of life while minimizing risks of overtreatment or undertreatment. These findings emphasize the importance of individualized care in this complex patient population and offer valuable insights for optimizing management strategies as the elderly demographic continues to grow.

## 1. Introduction

Breast cancer remains one of the most prevalent cancers among women globally, with approximately 12% of cases occurring in women aged 80 and older [1,2]. This trend, combined with an aging population, presents unique challenges for clinicians managing this subgroup.

Elderly patients demonstrate significant clinical heterogeneity, necessitating the careful consideration of factors such as biological age, pre-existing comorbidities, functional status, and life expectancy when tailoring treatment strategies.

Despite the increasing incidence of breast cancer in this age group, evidence-based guidelines specifically addressing the management of breast cancer in elderly patients remain scarce [3]. Standard protocols designed for younger populations—such as surgery, chemotherapy, and radiotherapy—are not always appropriate for older patients, as they may pose heightened risks of toxicities and complications. Furthermore, elderly women are often underrepresented in clinical trials, limiting the availability of robust data to guide personalized treatment plans [4,5].

This scenario underscores the critical importance of adopting a personalized approach to breast cancer management in elderly patients. Effective care must account not only for the cancer stage but also for the patient’s biological age, overall health status, comorbidities, and life expectancy.

A comprehensive geriatric assessment (GA) can play a pivotal role in stratifying patients into fit, prefrail, and frail categories, enabling clinicians to select the most appropriate treatment options for each group [6].

A multidisciplinary approach, integrating the expertise of oncologists, surgeons, and geriatric specialists, is essential to ensure individualized care. This approach can optimize treatment outcomes while minimizing the risks of overtreatment, which often leads to unnecessary morbidity and a diminished quality of life.

Balancing the potential benefits of aggressive therapies with their associated risks is crucial to improving both survival rates and overall patient well-being.

In light of the growing incidence of breast cancer in elderly women and the complexities inherent in their care, we conducted a retrospective study to evaluate clinical outcomes and treatment strategies for patients aged 80 and over treated at our institution.

The primary objective of this study is to offer valuable insights into the real-world management of breast cancer in this demographic, assess the impact of multidisciplinary care, and identify factors influencing survival and quality of life. By sharing these findings, we aim to enhance the understanding of breast cancer care in elderly patients and support the development of more personalized, evidence-based treatment strategies.

## 2. Materials and Methods

This retrospective study was conducted at our institution to analyze the clinical records of patients aged 80 years and older diagnosed with breast cancer between January 2016 and December 2020. The primary aim was to evaluate treatment strategies, clinical outcomes, and complications within this elderly cohort, with a particular focus on survival rates and the impact of a multidisciplinary care approach on treatment optimization.

### 2.1. Patient Selection

We identified 238 patients aged 80 or older who were referred to our breast cancer center with a new diagnosis of breast cancer during the study period. All patients had histologically confirmed breast cancer, diagnosed through either core needle biopsy or excisional biopsy, and were treated with curative intent. Patients with metastatic breast cancer at the time of diagnosis, as well as those with incomplete medical records, were excluded.

### 2.2. Clinical and Pathological Data

Clinical data collected for each patient included demographic information (age, comorbidities, performance status), clinical presentation (tumor size, stage, histological subtype), and results from initial diagnostic imaging (mammography, ultrasound, MRI). Disease staging followed the American Joint Committee on Cancer (AJCC) 8th edition system. Pathological data, including the presence of hormone receptors and immunophenotype, were available for a total of 241 lesions, as three patients had bilateral tumors.

### 2.3. Multidisciplinary Approach

A multidisciplinary team of oncologists, surgeons, radiologists, and geriatric specialists made treatment decisions. The treatment plan (surgery, adjuvant therapy, or palliative care) was based on factors such as the patient’s functional status, comorbidities, life expectancy, staging, and tumor biology.

Surgical options included breast-conserving surgery (BCS) or mastectomy, depending on tumor size, location, and patient preference. Axillary surgery, including sentinel lymph node biopsy or axillary dissection, was performed based on clinical and radiological findings [7]. Adjuvant therapies—radiotherapy, endocrine therapy, and chemotherapy—were prescribed according to tumor characteristics and the patient’s fitness level. Radiotherapy was administered to patients with node-positive disease or large tumors, while endocrine therapy (tamoxifen or aromatase inhibitors) was given to those with hormone receptor-positive breast cancer. Chemotherapy was considered for high-risk patients (HER2-positive, triple-negative) following an onco-geriatric assessment.

### 2.4. Postoperative Oncogeriatric Assessment

A Comprehensive Geriatric Assessment (CGA) was performed for patients with histologically confirmed early-stage breast cancer (stage 0-III, TNM classification) following SIOG guidelines and multidisciplinary tumor board discussion [8,9]. Weekly patient selection was based on registry criteria with exclusion of those with a life expectancy of less than six months or who refused participation. Life expectancy was calculated using the ePrognosis-Lee Schonberg Index tool.

The CGA included a thorough medical history, physical examination, and collection of socio-demographic data, comorbidities, oncological history, and relevant pathology/immunohistochemical information. Anthropometric data (weight, height, BMI) were also recorded [10,11,12]. As per SIOG and national guidelines, CGA components included performance status (ECOG scale), frailty assessment (Fried criteria), comorbidity burden (Charlson Comorbidity Index), functional status (Katz ADL, Lawton IADL), cognitive function (Mini-Mental State Examination), nutritional status (Mini Nutritional Assessment), mood (Geriatric Depression Scale), and physical performance (Short Physical Performance Battery) [13,14,15,16,17,18] (see Table 1).

Additional evaluations included screening for common geriatric syndromes (e.g., falls, incontinence) and social support factors. Frailty was defined according to Balducci’s criteria based on deficits in two or more CGA domains [20,21]. CGA results were discussed by the multidisciplinary team (breast surgeons, radiotherapists, and oncologists) to tailor individualized treatment plans with adjuvant therapy options based on NCCN guidelines [22].

### 2.5. Follow-Up and Outcomes

Following adjuvant treatments, all patients were regularly followed up with clinical examinations, mammography, and other imaging as needed. The follow-up period was defined as the time from surgery to the most recent visit or death. The main clinical outcomes assessed included overall survival (OS), disease-free survival (DFS), cancer-specific survival (CSS), and complications. Complications were categorized as surgical (e.g., wound dehiscence, hematoma) or non-surgical (e.g., lymphedema, chemotherapy-related toxicities). Twenty-nine patients were excluded due to loss to follow-up; attempts to contact them by phone or email were unsuccessful, likely due to technological barriers and a lack of alternative contact information.

### 2.6. Statistical Analysis

Descriptive statistics were used to summarize demographic and clinical data. Survival outcomes (OS, DFS, and CSS) were analyzed using Kaplan–Meier survival curves, with group differences assessed using the log-rank test. All statistical analyses were conducted using SPSS version 26.0 (IBM Corp, Armonk, NY, USA). A *p*-value of <0.05 was considered statistically significant.

### 2.7. Ethical Considerations

The study was approved by the Ethics Committee of “Fondazione Policlinico Universitario Agostino Gemelli IRCCS” in Rome (number 5357). All patient data were anonymized prior to analysis to ensure confidentiality. As a retrospective chart review, informed consent was not required.

## 3. Results

A total of 238 patients aged 80 years and older, diagnosed with breast cancer between January 2016 and December 2020, were included in this retrospective study. The median age of the cohort was 84 years (range 80–97 years). Of these, 29 patients (12%) were lost to follow-up, and survival outcomes were assessed for the remaining 209 patients.

Among the cohort, 5 patients (2%) were male, and 3 patients (1.2%) had bilateral tumors. Invasive carcinomas were diagnosed in 232 patients (97.5%), while 5 patients (2.1%) had ductal carcinoma in situ (DCIS), and 1 patient (0.4%) had DCIS with suspected stromal microinvasion. Of the 238 patients, 203 (85.3%) had no suspicious lymph nodes at diagnosis, 32 (13.4%) were N1, 2 (0.8%) were N2, and 1 patient (0.4%) was N3.

Tumor characteristics are summarized in Table 2.

Histopathological examination revealed invasive ductal carcinoma in 161 patients (67.6%), invasive lobular carcinoma in 40 patients (16.8%), and other histologies (e.g., mucinous, metaplastic, papillary) in 29 patients (12.1%). Tumor grade was available for 230 cases, with 133 tumors (57.8%) classified as intermediate grade (G2), 77 (33.4%) as high grade (G3), and 17 (7.3%) as low grade (G1). Hormone receptor positivity was found in 204 tumors (84.6%), with 52% categorized as Luminal A-like and 48% as Luminal B-like. HER2 positivity was detected in 16 tumors (6.6%), and 20 tumors (8.3%) were triple-negative. Three lesions (1.2%) were not classifiable due to equivocal FISH results, phyllodes tumor, and invasive solid papillary neoplasia.

Of the 238 patients, 203 (85.3%) underwent breast-conserving surgery (BCS), and 35 (14.7%) underwent mastectomy due to factors such as tumor size, location, or patient preference. Axillary surgery was performed in 129 patients (54.2%), with the majority (57.3%) undergoing sentinel lymph node biopsy.

Adjuvant treatments were personalized based on tumor biology, staging, and patient health. Ninety-three patients (39.1%) received adjuvant radiotherapy, in accordance with international guidelines (BCS or mastectomy for stage >pT2 or nodal-positive disease). Endocrine therapy was administered to 101 patients (42.4%), primarily those with hormone receptor-positive tumors. Chemotherapy was considered for high-risk patients following a comprehensive onco-geriatric assessment and was administered to 6 patients (2.5%), including 4 with HER2-positive tumors and 2 with triple-negative tumors. These patients received anthracycline-free chemotherapy without reported toxicity, highlighting the potential for tailored therapy even in elderly individuals. Thirty-eight patients (16%) received no adjuvant treatment due to clinical or psychological frailty or personal choice. Treatment data are summarized in Table 3.

### 3.1. Oncological Outcomes

The median follow-up time for the entire cohort was 42.3 months.

- Local recurrence: One patient (0.5%) experienced a local relapse after surgery;
- Regional recurrence: One patient (0.5%) had regional node recurrence;
- Distant metastasis: Nineteen patients (9%) developed distant metastases, most commonly in the bone and lungs;
- Breast cancer-related mortality: Nineteen patients (9%) died due to breast cancer progression.

The survival outcomes were as follows:

- Disease-free survival (DFS): 66.6% at 5 years (see Figure S1);
- Cancer-specific survival (CSS): 88.5% at 5 years (see Figure S2);
- Overall survival (OS): 73.3% at 5 years (see Figure S3).

### 3.2. Postoperative Complications

The overall complication rate was 10%. The most common complications were fluid collection (5.9%), wound dehiscence (2.1%), and hematoma (0.4%) (see Table 4).

### 3.3. Chemotherapy-Related Toxicities

No chemotherapy-related toxicities were reported among the small subset of patients who received chemotherapy. These results suggest that multidisciplinary management in elderly breast cancer patients can yield favorable outcomes with relatively low morbidity [23,24,25].

## 4. Discussion

Breast cancer in elderly patients, particularly those aged 80 years and older, presents unique challenges in clinical management due to the complexity of their clinical profiles. This study aimed to evaluate the treatment strategies and clinical outcomes of breast cancer in this age group, emphasizing the role of multidisciplinary care and onco-geriatric assessments in optimizing outcomes and minimizing both over-treatment and under-treatment. The results suggest that breast cancer in elderly patients can be effectively managed through a tailored approach that balances oncological efficacy with the patient’s quality of life.

### 4.1. Surgical Management

Treatment decisions for breast cancer in elderly patients must be individualized, taking into account functional status, comorbidities, and life expectancy. In this study, 85.3% of patients underwent BCS while 14.7% required mastectomy, often due to factors such as tumor size or location. These findings are consistent with previous studies showing that BCS is safe and effective for elderly women with early-stage breast cancer, offering advantages like better cosmetic outcomes and reduced morbidity compared to mastectomy. However, mastectomy remains the preferred option for larger tumors or when there is a significant risk of local recurrence.

Axillary surgery was performed in 54% of patients, with 31.1% undergoing sentinel lymph node biopsy (SLNB) and 12.6% undergoing axillary dissection (AD) due to clinically positive lymph nodes at diagnosis or during intraoperative exploration. In 46% of cases, axillary evaluation was not performed based on preoperative multidisciplinary team (MDT) recommendations. These findings align with current guidelines that advocate for SLNB in patients with clinically node-negative disease. Our data confirm that axillary surgery can be safely performed in elderly patients when part of a well-coordinated, multidisciplinary treatment plan that includes geriatric assessments [9,26].

### 4.2. Postoperative Histopathological Findings

Postoperative histopathology showed that among the 203 patients who underwent BCS, 5.4% had pTis tumors, 53.7% had pT1 tumors, 39% had pT2 tumors, and 1% had pT3 tumors. Multifocal disease was present in 4.4% of cases. Among the 35 patients who underwent mastectomy, 17 had pT2 tumors, 12 had pT3 or pT4 tumors, and 6 had pTis or pT1 tumors. Of the 129 patients who received axillary surgery, 62.8% had pN0 (node-negative) status, while 25.6% were pN1, 7.8% were pN2, and 3.8% were pN3 (see Table 5).

### 4.3. Adjuvant Treatment and Onco-Geriatric Assessments

Adjuvant therapy plays a crucial role in reducing the risk of recurrence and improving survival in breast cancer patients. In our study, 39.1% of patients received adjuvant radiotherapy, and 42.4% received endocrine therapy. These treatment strategies align with current clinical guidelines for elderly breast cancer patients. The decision to administer adjuvant therapy in elderly patients is influenced by tumor biology, patient health, and functional status. Radiotherapy was primarily given to patients who underwent BCS and had node-positive or high-risk tumors, reflecting its role in local control post-surgery [27,28,29].

Endocrine therapy was widely used in this cohort, with 42.4% of patients receiving it. This reflects the high proportion of hormone receptor-positive tumors in elderly patients. Hormone therapies like tamoxifen or aromatase inhibitors are cornerstones of treatment for hormone receptor-positive breast cancer, offering a favorable risk-to-benefit ratio even in older patients. The relatively high use of endocrine therapy in our cohort suggests that elderly patients can tolerate long-term hormonal treatment with minimal toxicity, provided they undergo proper assessments [30,31,32].

Chemotherapy was given to a small subset of high-risk patients after onco-geriatric evaluations, which represents a promising approach for managing elderly patients with high-risk disease. While chemotherapy can be effective, it poses a risk of significant toxicity in the elderly due to reduced organ function and increased comorbidities [33]. Notably, the six elderly patients who received chemotherapy in our study did not experience significant toxicity, indicating that, with appropriate monitoring and tailored treatment plans, chemotherapy can be safely administered to selected elderly patients.

### 4.4. Complications and Morbidity

Postoperative complications are an important consideration in elderly breast cancer patients who may be more prone to surgical issues due to frailty, poor nutritional status, and comorbidities. In our study, the overall complication rate was 10%, with wound dehiscence, hematoma, and lymphedema being the most common issues. These rates are consistent with those observed in other studies of elderly breast cancer patients. However, with preoperative optimization and geriatric assessments, the incidence of complications can be minimized.

Lymphedema, particularly in patients undergoing axillary surgery, is a concern. While the incidence of lymphedema in our cohort was relatively low, it remains a significant morbidity in this population. Future studies should explore strategies to prevent lymphedema, such as less invasive axillary procedures or early physiotherapy interventions.

### 4.5. Survival Outcomes

Elderly patients often experience worse survival outcomes due to age-related factors and comorbidities. In a retrospective Canadian study published in 2023, the authors studied a cohort of 24,469 patients diagnosed with breast cancer between 2005 and 2014 with age between 35 and over 80; they found that 5 years OS (overall survival) for patients over 80 years is 57.3% and CSS is 79.9% [34].

However, our study demonstrated favorable survival outcomes; in fact, 5-year overall survival (OS), disease-free survival (DFS), and cancer-specific survival (CSS) rates were 73.3%, 66.6%, and 88.5%, respectively.

We also stratified the overall survival curves by type of adjuvant treatment, noting that patients who underwent radiotherapy had worse survival compared to those who received no treatment. This can be explained by the fact that patients selected for radiotherapy had multifocal disease greater than 2 cm, some of them with nodal involvement (See Figure S4).

The low rates of local (0.5%) and regional recurrence (0.5%) further underscore the effectiveness of our treatment approach, which combined surgery with adjuvant therapies.

Despite these positive outcomes, the 5-year DFS of 66.6% indicates that a proportion of elderly patients still experience disease recurrence, highlighting the need for close follow-up and continued research to optimize treatments. The CSS of 88.5% reflects the effectiveness of modern therapies but also underscores the mortality risk from comorbid conditions, which must be considered when making treatment decisions [35,36,37].

### 4.6. Limitations and Future Directions

While this study offers valuable insights into the management of breast cancer in elderly patients, there are some limitations. Being a retrospective analysis, the study cannot establish causal relationships. Additionally, the small number of patients receiving chemotherapy may not fully represent the experience of elderly women with high-risk disease. Future prospective studies with larger, more diverse cohorts are necessary to confirm our findings and explore the role of novel treatments, such as targeted therapies and immunotherapy, in elderly breast cancer patients.

## 5. Conclusions

This study highlights the effectiveness of a personalized, multidisciplinary approach in managing breast cancer among elderly patients, particularly those aged 80 and above [38]. With comprehensive onco-geriatric assessments, treatments such as breast-conserving surgery, axillary surgery, and adjuvant therapies can be successfully implemented, even in a population with complex clinical profiles. Chemotherapy, when reserved for carefully selected high-risk patients, can be safely and effectively administered with minimal morbidity. Our findings demonstrate that survival outcomes in this elderly cohort are comparable to those observed in younger populations, emphasizing the importance of balancing oncological efficacy with the preservation of quality of life.

However, the results underline the urgent need for more robust evidence and the development of tailored clinical guidelines to support decision-making for elderly women with breast cancer. As the population continues to age, advancing personalized care strategies will be essential to address the unique challenges and opportunities in this patient group, ensuring optimal outcomes and minimizing treatment-related burdens.

## Figures and Tables

**Table 1:** Postoperative Oncogeriatric Assessment.

ECOG scale [11]	Points 0–4 0. Fully active 1. Restricted in physically strenuous activity; able to carry out work of a light or sedentary nature 2. Ambulatory; capable of all self-care; unable to carry out any work activities; up more than 50% 3. Capable of only limited self-care; confined to bed or chair for more than 50% of waking hours 4. Completely disabled; cannot carry out any self-care; totally confined to bed or chair
Fried Criteria [19]	Points 0–5 (Frail ≥ 3) Weight lost (>4.5 kg in the last year) (0: no; 1: yes) Fatigue (low energy sensation almost 3 days a week (0: no; 1: yes) Low muscle strength (handgrip < 3.37 kg) (0: no; 1: yes) Low physical activities (0: no; 1: yes) Low walking speed (<0.8 m/s) (0: no; 1: yes)
Charlson Comorbidity Index [13]	Points 0–33 (cumulative comorbidity index) Age (0: <50; 1: 50–59; 2: 60–69; 3: 70–79; 4: >80) Myocardial infarction (0: no; 1: yes) Cardiac failure (0: no; 1: yes) Peripheral artery disease (0: no; 1: yes) Stroke (0: no; 1: yes) Dementia (0: no; 1: yes) COPD (0: no; 1: yes) Connective disease (0: no; 1: yes) Peptic ulcer (0: no; 1: yes) Hepatic disease (0: no; 1: mild: 3: moderate/severe) Diabetes (0: no; 1: uncomplicated: 2: complicated) Hemiplegia (0: no; 2: yes) Renal Failure (0: no; 2: yes) Solid tumors (0: no; 2: localized; 6 metastatic) Leukemia (0: no; 2: yes) Linphoma (0: no; 2: yes) AIDS (0: no; 6: yes)
ADL [14]	0–6 (0: totally dependent; 6: independent) Bathing (0: dependent; 1: independent) Dressing (0: dependent; 1: independent) Eating (0: dependent; 1: independent) Toileting (0: dependent; 1: independent) Transferring (0: dependent; 1: independent) Continence (0: incontinent; 1: continent)
IADL [15]	0–8 (0: totally dependent; 8: independent) Using the telephone (0: dependent; 1: independent) Shopping (0: dependent; 1: independent) Preparing food (0: dependent; 1: independent) Housekeeping (0: dependent; 1: independent) Doing laundry (0: dependent; 1: independent) Using transportation (0: dependent; 1: independent) Handling medications (0: dependent; 1: independent) Handling finances (0: dependent; 1: independent)
MMSE [16]	0–30 (Cognitive problems < 24)
MNA [17]	0–30 (24–30: normal; 17–23.5 risk of malnutrition; <17 poor nutritional status
GDS-15 [18]	0–15 (normal 3 ± 2; mild depression 7 ± 3; very depressed 12 ± 2)

ADL: activities of daily living; IADL: instrumental activities of daily living; MMSE: mini-mental state examination; MNA: mini nutritional assessment; GDS: geriatric depression scale.

**Table 2:** Patients’ and tumors’ characteristics.

AGE (median, IQR, range)	84, 81–86, 80–97
GENDER	
M	5 (2.1)
F	233 (97.9)
cT	
in situ	5 (2.1)
x	1 (0.4)
1a	11 (4.6)
1b	39 (16.4)
1c	65 (27.3)
2	96 (40.3)
3	8 (3.4)
4	9 (3.8)
4a	1 (0.4)
4b	2 (0.8)
4d	1 (0.4)
cN	
0	203 (85.3)
1	32 (13.4)
2	2 (0.8)
3	1 (0.4)

**Table 3:** Treatment strategies.

NAD	
No	192 (80.7)
HT	44 (18.5)
CT	2 (0.8)
SURGERY (T)	
Q	203 (85.3)
M	35 (14.7)
SURGERY (N)	
None	109 (45.8)
LS	74 (31.1)
Sampling	25 (10.5)
LA	30 (12.6)

NAD (neoadjuvant), HT (Hormone therapy), CT (Chemotherapy), Q (Quadrantectomy), M (Mastectomy), M+R (Mastectomy plus reconstruction), LS (Sentinel lymph node), LA (Lymphadenectomy).

**Table 4:** Adjuvant treatment and post-surgical complications.

ADJUVANT TREATMENT	
None	38 (16.0)
CT	5 (2.1)
CT+RT	1 (0.4)
OT alone	101 (42.4)
RT alone	15 (6.3)
RT+OT	78 (32.8)
COMPLICATIONS	
Dehiscence	5 (2.1)
Hematoma	1 (0.4)
Liquid collect	14 (5.9)
Lymphedema	3 (1.3)
Other (trombosis)	1 (0.4)

**Table 5:** Histopathological findings.

pT	
in situ	11 (4.6)
0	2 (0.8)
1a	9 (3.8)
1b	24 (10.1)
1c	76 (31.9)
1mic	2 (0.8)
2	94 (39.5)
3	7 (2.9)
4	6 (2.5)
4a	1 (0.4)
4b	4 (1.7)
4c	1 (0.4)
other	1 (0.4)
pN	
0	81 (62.8)
1	33 (25.6)
2	10 (7.8)
3	5 (3.8)
IMMUNOPHENOTYPE	
Luminal A	106 (52)
Luminal B	96 (47)
TN	20 (8.3)
Her2	16 (6.6)
Other	3 (1.2)

## Data Availability

The original contributions presented in this study are included in the article/supplementary material. Further inquiries can be directed to the corresponding author.

## Supplementary Materials

The following supporting information can be downloaded at https://www.mdpi.com/article/10.3390/jpm15030090/s1, Figure S1: Disease-Free Survival; Figure S2: Cancer-Specific Survival; Figure S3: Overall Survival; Figure S4: Overall survival stratified by type of adjuvant treatment.
