# Supplementary material for: Advancing Breast Cancer Care in Patients Aged 80 and Above: A Personalized and Multidisciplinary Management to Better Outcomes
Source: J Pers Med. 2025 Feb 27;15(3):90. doi: 10.3390/jpm15030090 (PMC11943859; doi:10.3390/jpm15030090)
Supplement: Supplementary file 1 [file jpm-15-00090-s001.zip › jpm-3385655-supplementary.pdf]

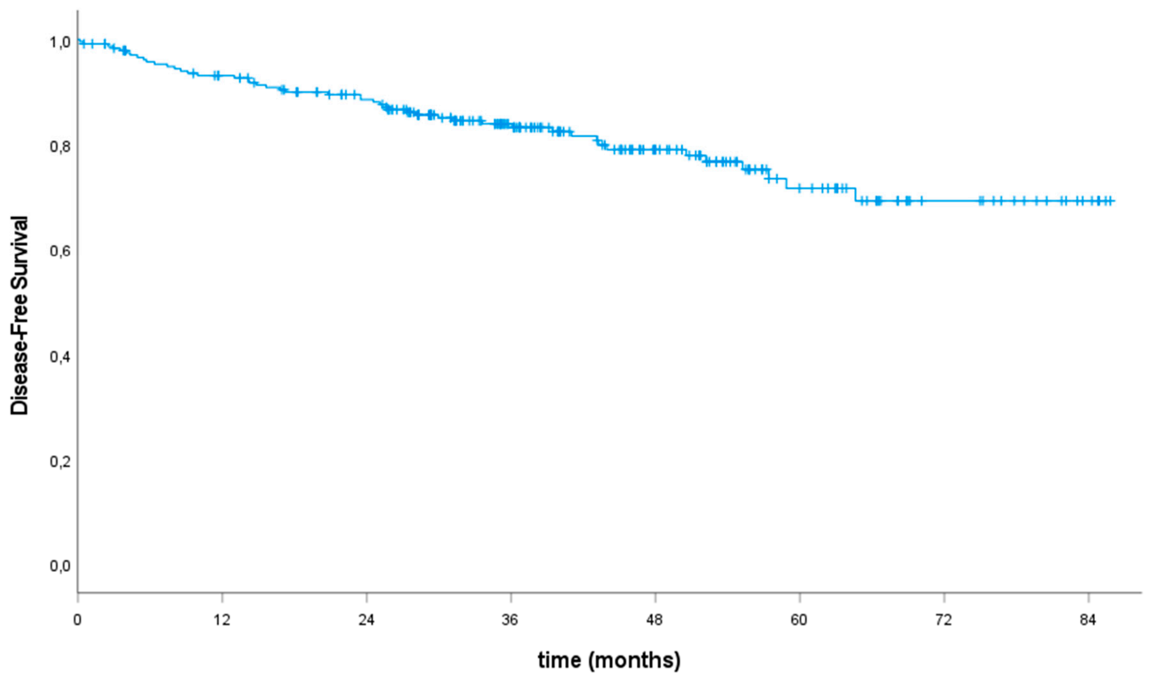

Figure S1. Disease-Free Survival

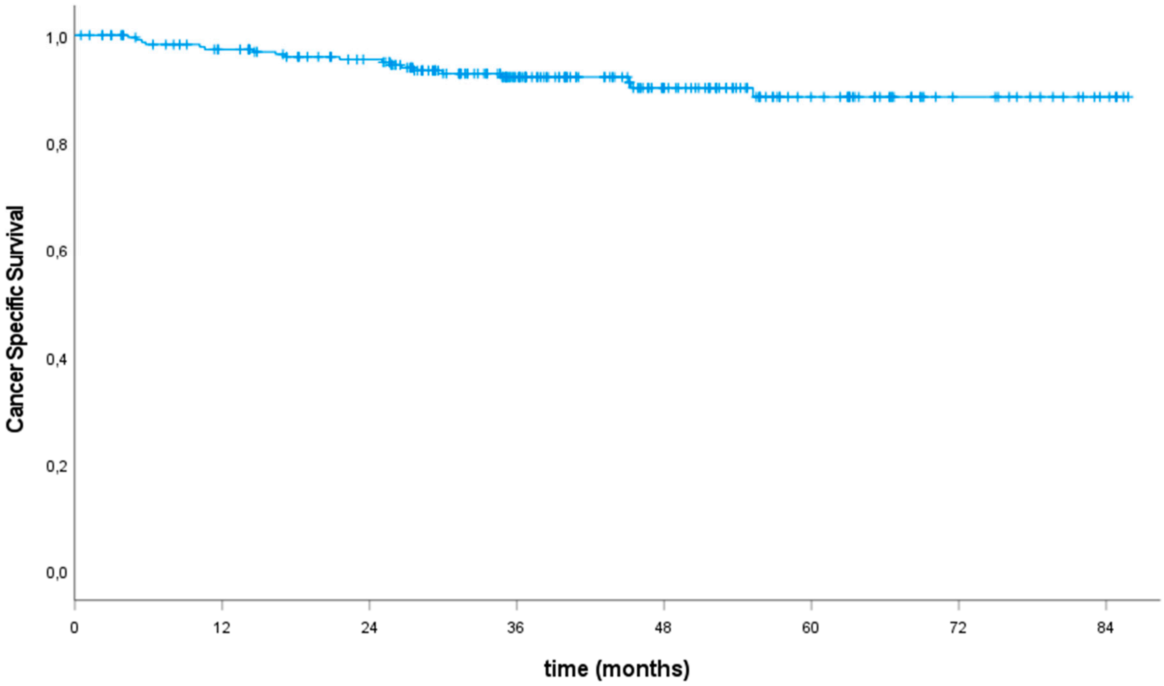

Figure S2. Cancer Specific Survival

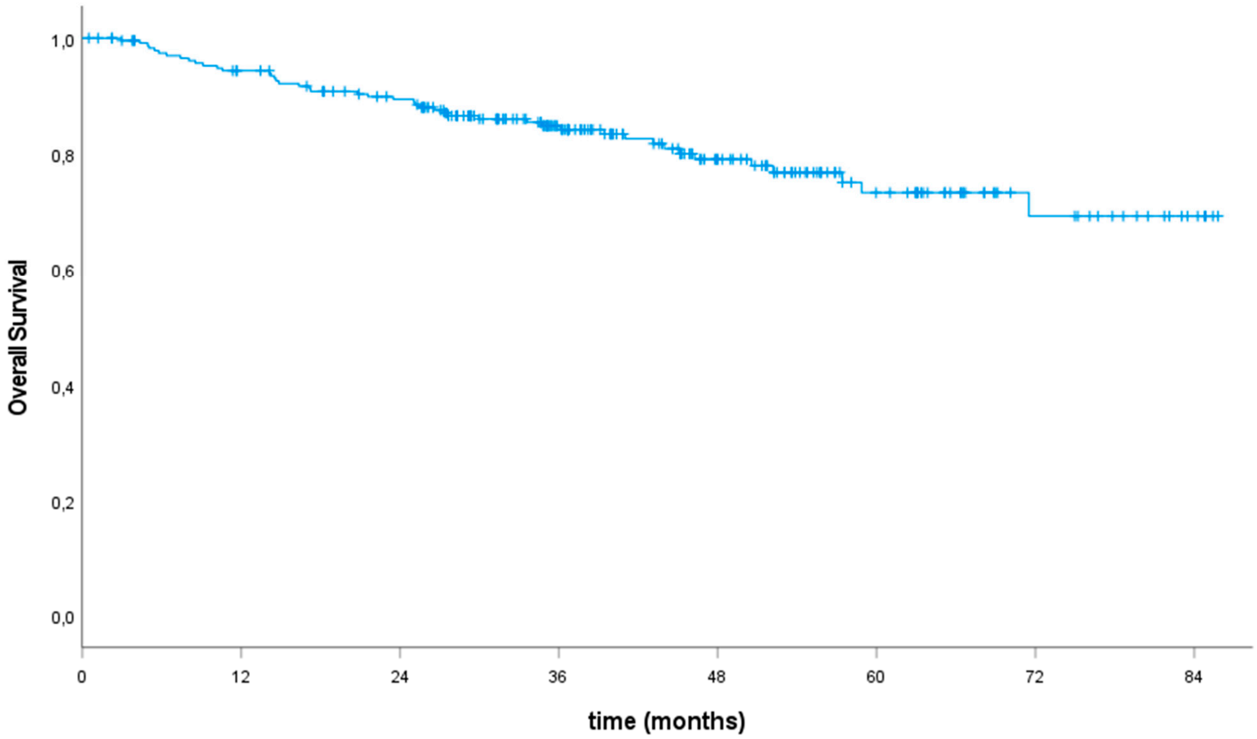

Figure S3. Overall Survival

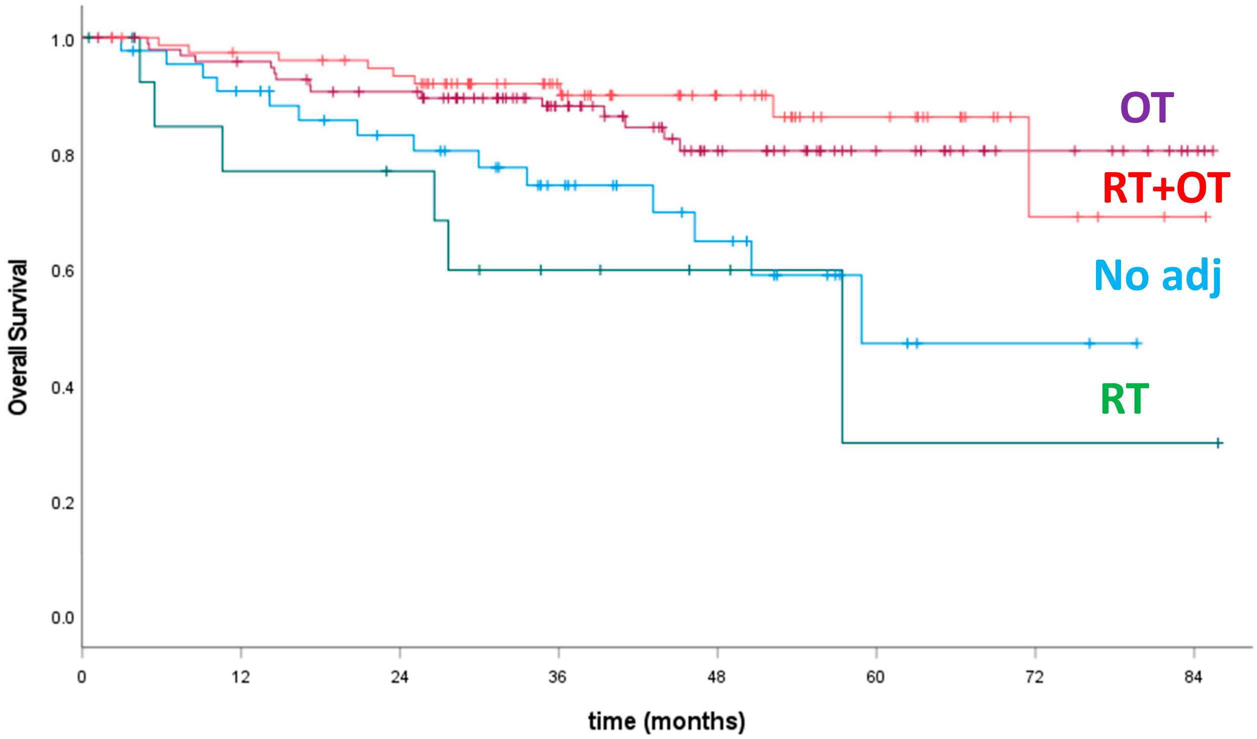

Figure S4. Overall survival stratified by type of adjuvant treatment
